# Supplementary material for: On-Demand Release of Fucoidan from a Multilayered Nanofiber Patch for the Killing of Oral Squamous Cancer Cells and Promotion of Epithelial Regeneration
Source: J Funct Biomater. 2022 Sep 28;13(4):167. doi: 10.3390/jfb13040167 (PMC9589995; doi:10.3390/jfb13040167)
Supplement: Supplementary file 1 [file jfb-13-00167-s001.zip › jfb-1937722-supplementary.pdf]

*Supplemental Information*

# On-Demand Release of Fucoidan from a Multilayered Nanofiber Patch for the Killing of Oral Squamous Cancer Cells and Promotion of Epithelial Regeneration

Yingnan Liu [1,†], Yingjie Xu [2,†], Xiaopei Zhang [1], Na Liu [1], Beibei Cong [2], Yu Sun [2], Mingxia Guo [1], Zeyu Liu [1], Le Jiang [1], Wanchun Wang [2], Tong Wu [1,3,*] and Yuanfei Wang [2,*]

[1] Institute of Neuroregeneration and Neurorehabilitation, Qingdao Medical College, Qingdao University, Qingdao 266071, China; yingnanliu2021@126.com (Y.L.); xp_dreamfly@163.com (X.Z.); a1349747636@163.com (N.L.); pipi_pixie@163.com (M.G.); liuzeyuqddx@163.com (Z.L.); 15215346351@163.com (L.J.)

[2] Qingdao Stomatological Hospital, Qingdao 266001, China; xyjdywe@163.com (Y.X.); xinruo1986@163.com (B.C.); sunyumed@163.com (Y.S.); kqwwch@126.com (W.W.)

[3] Shandong Key Laboratory of Medical and Health Textile Materials, Collaborative Innovation Center for Eco-Textiles of Shandong Province and the Ministry of Education, 308 Ningxia Road, Qingdao 266071, China

* Correspondence: twu@qdu.edu.cn (T.W.); zhizunbao19@163.com (Y.W.)

† These authors contributed equally to this work.

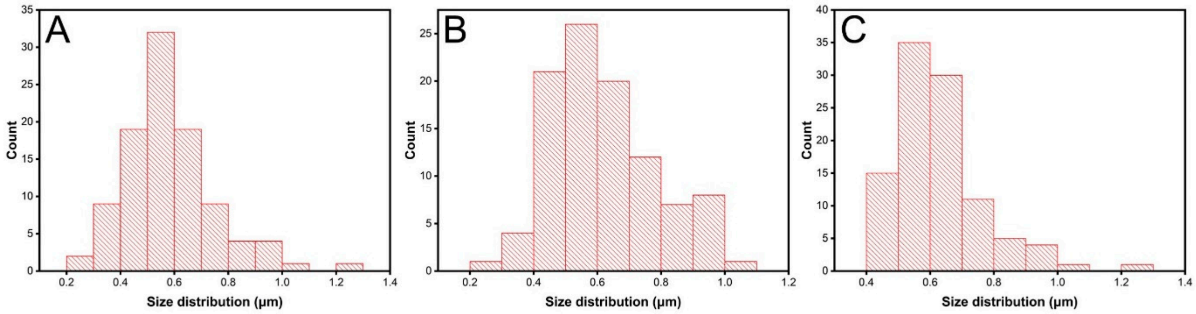


**Figure S1** The diameter distribution of (A) PCM nanoparticles, (B) PCL nanofibers, and (C) PCL/ADM nanofibers.

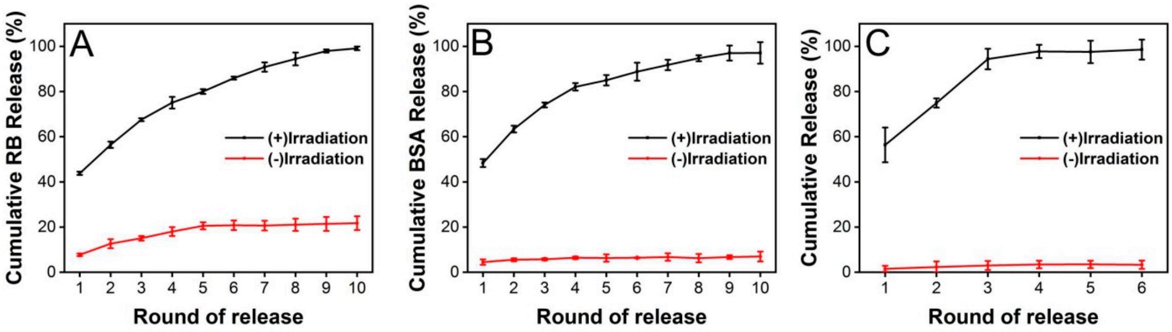


**Figure S2** Cumulative release profiles of different payloads released from the PCM microparticles. (A) RB, (B) FITC-BSA, and (C) LMWF released from PCMs loaded with ICG (1.0 mg/mL) under an NIR laser at an irradiation density of 2600 mW (n=3). The time interval of laser irradiation between two adjacent cycles was 0.5 h.

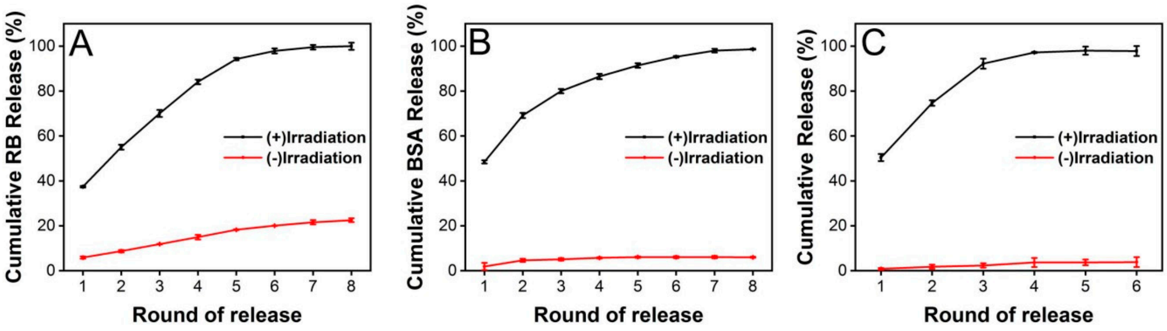


**Figure S3** Cumulative release profiles of different payloads released from the PCM microparticles. (A) RB, (B) FITC-BSA, and (C) LMWF released from PCMs loaded with ICG (1.0 mg/mL) under an NIR laser at an irradiation density of 2600 mW (n=3). The time interval of laser irradiation between two adjacent cycles was 2.0 h.

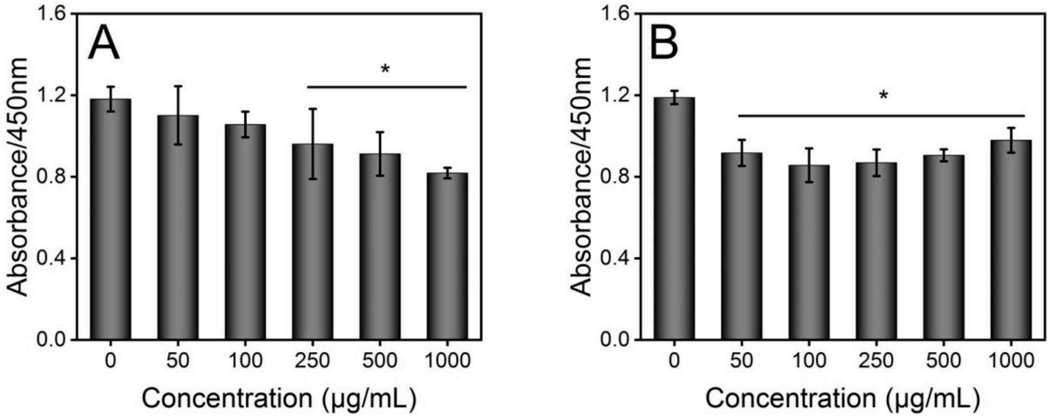


**Figure S4** (A) The cytotoxicity of different concentrations of LMWF toward HOK cells. No obvious cytotoxicity was observed until the concentration reached 250 μg/mL. (B) The effective concentrations of LMWF toward SCC-9 cells. The LMWF exhibited significant cytotoxicity toward SCC-9 cells ranging from 50 to 1000 μg/mL. * $P<0.05$ when comparing the groups with groups without LMWF treatment (0 μg/mL).

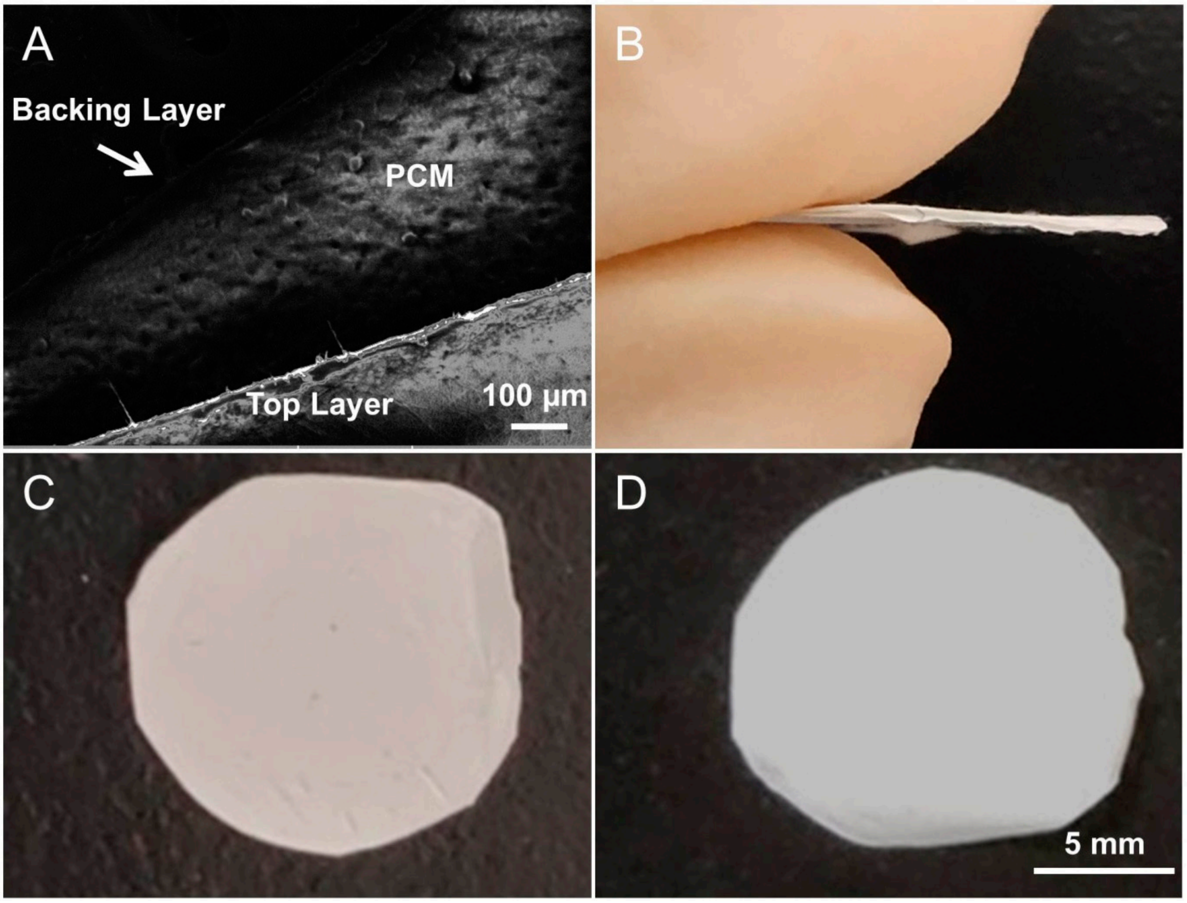


**Figure S5** (A) The SEM image of the multi-layered scaffold. (B-D) The photograph of the multi-layered scaffold (B) The sectional view of the multi-layered scaffold. (C) The front view of the multi-layered scaffold. (D) The back view of the multi-layered scaffold.

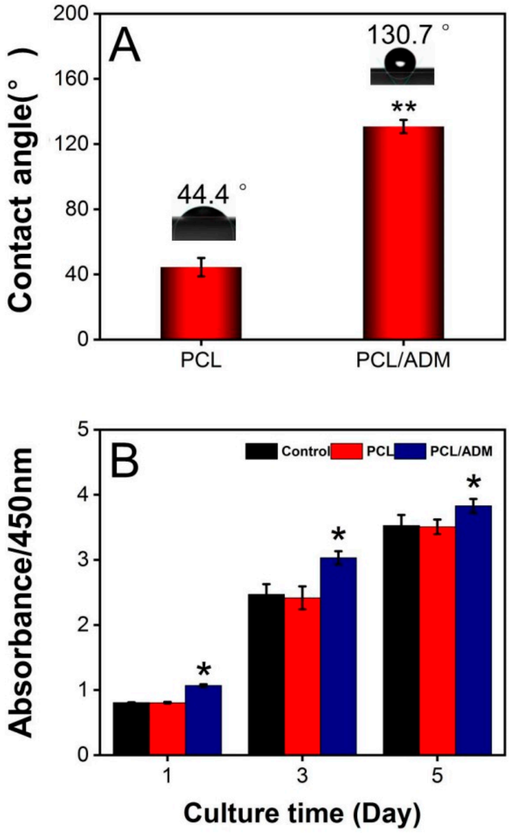


**Figure S6** (A) Water contact angle assay for the various fibers bythe sessile drop technique. (n=3). ** *P*<0.01 when comparing the group of PCL/ADM fibers with the other group. (B) The comparison of cell proliferation of HOK cells cultured on PCL and PCL/ADM fibers (n=3). * *P*<0.05 when comparing the group of PCL/ADM fibers with other groups.

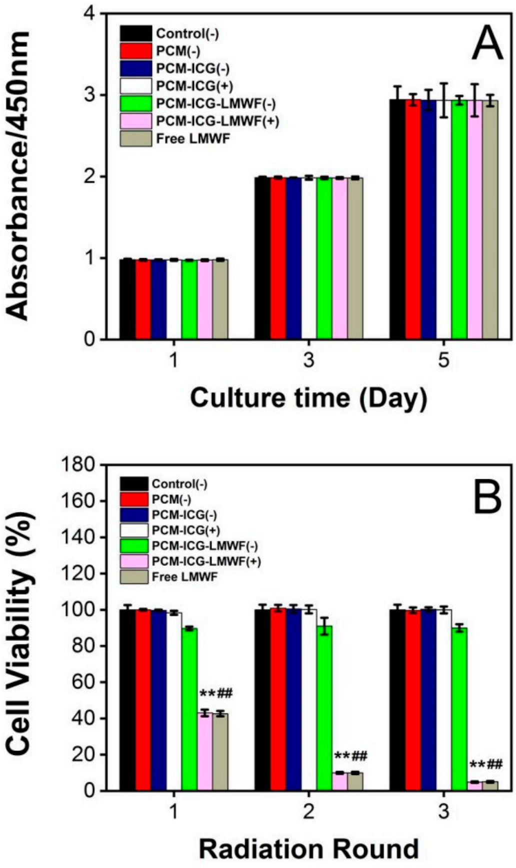


**Figure S7** Cell viability of various types of PCMs toward (A) HOK cells and (B) SCC-9 cell lines under 60s irradiation(2600mW) or without irradiation at pre-appointed time points during culturing for five days. "+" indicated the irradiation treatment, and "-" indicated without irradiation treatment. The Free LMWF group means the addition of an equivalent dosage of LMWF at time points (n=3). ** $P<0.01$ when comparing the group of PCM-ICG-LMWF (+) PCM particles under irradiation with other groups. ## $P<0.01$ when comparing the group of Free LMWF with other groups.

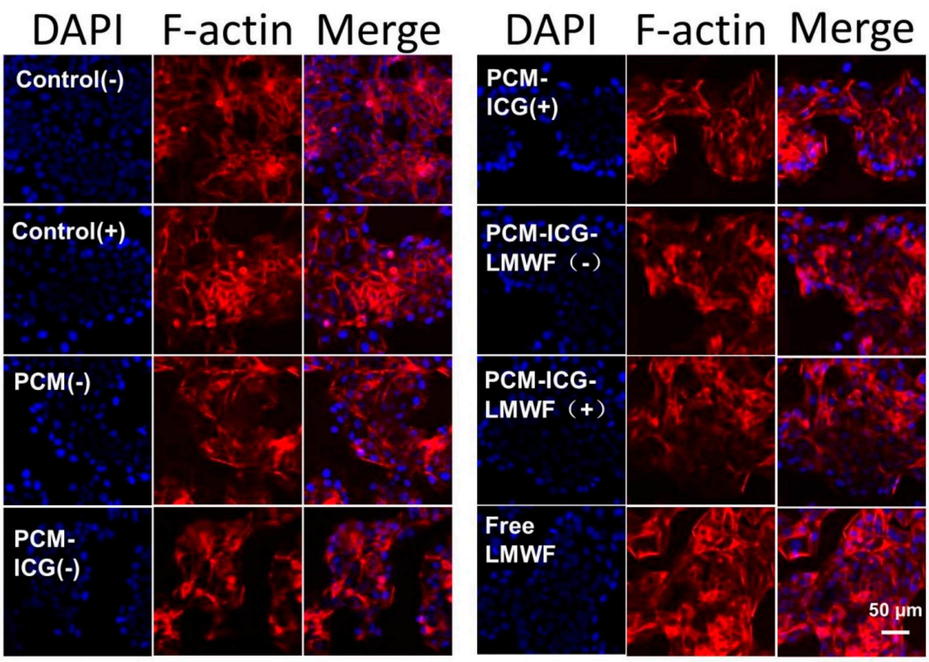


**Figure S8** Fluorescence images of HOK cell morphology incubated with various types of multi-layered scaffolds under 60s irradiation (2600mW) or without irradiation at pre-appointed time points during culture for five days. The Free LMWF group means the addition of an equivalent dosage of LMWF at time points. "+" indicated the irradiation treatment, and "-" indicated without irradiation treatment (n=3).

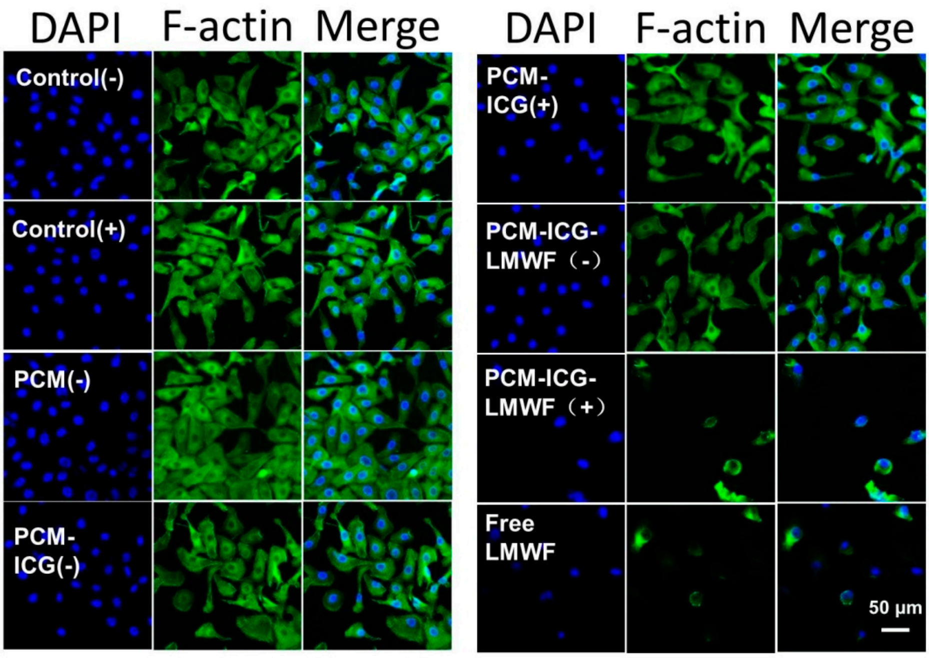


**Figure S9** Fluorescence images of SCC-9 cell morphology incubated with various types of multi-layered scaffolds under 60s irradiation (2600mW) or without irradiation at pre-appointed time points during culture for five days. The Free LMWF group means the addition of an equivalent dosage of LMWF at time points. "+" indicated the irradiation treatment, and "-" indicated without irradiation treatment (n=3).
